# Association of interleukin-2 and interleukin-10 with the pathophysiology and development of generalized anxiety disorder: a case-control study

**DOI:** 10.1186/s12888-024-05911-z

**Published:** 2024-06-20

**Authors:** Nisat Sarmin, A. S. M. Roknuzzaman, Rapty Sarker, Mamun -or-Rashid, MMA Shalahuddin Qusar, Sitesh Chandra Bachar, Eva Rahman Kabir, Md. Rabiul Islam, Zobaer Al Mahmud

**Affiliations:** 1 Department of Clinical Pharmacy and Pharmacology, Faculty of Pharmacy, University of Dhaka, Dhaka, 1000 Bangladesh; 2 Department of Pharmacy, University of Asia Pacific, Dhaka, 1205 Bangladesh; 3 Department of Psychiatry, Bangabandhu Sheikh Mujib Medical University, Shahabagh, Dhaka, 1000 Bangladesh; 4 Department of Pharmacy, Faculty of Pharmacy, University of Dhaka, Dhaka, 1000 Bangladesh; 5 School of Pharmacy, BRAC University, Kha 224 Bir Uttam Rafiqul Islam Avenue, Merul Badda, Dhaka, 1212 Bangladesh

**Keywords:** Generalized anxiety disorder, Mental disorders, Interleukin-2, Interleukin-10, Cytokines, Pathology

## Abstract

**Background:**

Generalized anxiety disorder (GAD) is a devastating mental health condition characterized by constant, uncontrolled worrying. Recent hypotheses indicate that pro-inflammatory cytokines and chemokines are potential contributors to the pathogenesis of GAD. Here, we aimed to assess the role of interleukin-2 (IL-2) and interleukin-10 (IL-10) in the pathophysiology and development of GAD.

**Methods:**

This study recruited 50 GAD patients diagnosed according to the DSM-5 criteria and 38 age-sex-matched healthy controls (HCs). A qualified psychiatrist evaluated all study subjects. The socio-demographic and clinical characteristics of the study population were determined using pre-structured questionnaires or interviews, and cytokine serum levels were estimated using commercially available ELISA kits.

**Results:**

We observed reduced serum IL-10 levels in GAD patients compared to HCs (33.69 ± 1.37 pg/ml vs. 44.12 ± 3.16 pg/ml). Also, we observed a significant negative correlation between altered IL-10 levels and GAD-7 scores (*r*=-0.315, *p* = 0.039). Moreover, IL-10 serum measurement exhibited good predictive value in receiver operating characteristics (ROC) analysis with an area under the curve (AUC) value of 0.793 (*p* < 0.001) with 80.65% sensitivity and 62.79% specificity at a cutoff value of 33.93 pg/ml. Conversely, we noticed elevated serum IL-2 levels in GAD patients than in HCs (14.81 ± 2.88 pg/ml vs. 8.08 ± 1.1 pg/ml); however, it failed to maintain any significant association with GAD-7 scores, implying that IL-2 might not be involved in GAD pathogenesis. The lower AUC value (0.640; *p* > 0.05) exhibited by IL-2 serum measurement in ROC analysis further supported that IL-2 might not be associated with GAD.

**Conclusion:**

This study provides new insights into the complex interplay between anti-inflammatory cytokines and GAD pathogenesis. Based on the present findings, we can assume that IL-10 but not IL-2 may be associated with the pathophysiology and development of GAD. However, further research with a larger population size and longitudinal design is required to confirm the potential diagnostic efficacy of IL-10.

## Background

Generalized anxiety disorder (GAD) is a chronic neuropsychiatric disorder characterized by persistent and excessive uncontrollable fear or worry (occurs for at least 6 months) about various aspects/activities of daily life, affecting the educational, occupational, or social lives of the affected people [1]. If a person is excessively worried about anything for most days over at least 6 months, he/she is considered to have GAD. Though currently the prevalence rate of GAD is 3–6% worldwide [1–3], the prevalence is increasing day by day due to the complexity of modern lifestyles and thus warrants attention from national and international authorities to take interventions for mitigating and managing this disorder properly. If it remains undiagnosed or untreated, the uncontrollable and persistently intense anxiety can lead to a marked reduction in cognitive functions or a reduced capacity to work properly in all spheres of life, including educational, family, social, and individual routine work. As such, chronic GAD leads to a reduced quality of life and thereby poses a significant mental health concern globally.

Despite its high prevalence, significant morbidity, and socioeconomic burden, GAD remains poorly characterized in terms of its pathophysiology or effective treatment options. Though the precise cause and mechanism of pathogenesis are still unknown, evidence suggests that multiple factors, including disrupted serotonergic, dopaminergic, and GABAergic neurotransmission and excessive glutamatergic neurotransmission in the brain, genetic factors, family or environmental stress, chronic diseases, hyperthyroidism, childhood trauma, and special personality traits, are linked to GAD. Alterations in monoaminergic neurotransmissions in limbic systems (cingulate gyrus, hippocampus, amygdala, thalamus, and hypothalamus) due to the lower synaptic availability of serotonin, norepinephrine, and dopamine are thought to be associated with anxiety symptoms. Besides, decreased GABA-mediated inhibitory neurotransmission in the amygdala or excessive activation of excitatory glutamatergic neurotransmission are also suggested to be involved in GAD pathology.

Currently, available pharmacotherapies for GAD include selective serotonin reuptake inhibitors (SSRIs), serotonin and norepinephrine reuptake inhibitors (SNRIs), pregabalin, and benzodiazepines, which act by reversing these altered monoaminergic neurotransmitter systems. Alongside these drug treatments, non-pharmacological therapies such as several psychological interventions, including cognitive-behavioral therapy, and the acquisition and application of stress management skills, including relaxation and mindfulness skills are also widely used for the management of GAD. However, currently, available pharmacotherapies (SSRIs, SNRIs, pregabalin, and benzodiazepines) have failed to demonstrate the required efficacy in treating anxiety disorders, as 50% of patients failed to respond to these drugs, and at least in 30% of cases, there is a recurrence of the disease following the pharmacological treatment [1, 4, 5]. Moreover, studies reported a higher rate of discontinuity from these pharmacotherapies with low patient adherence or compliance due to the adverse effects, including sexual dysfunction for SSRIs and SNRIs, nausea and dizziness for pregabalin, demonstrating an urgent need for searching for novel anxiolytics [3]. These findings raised questions about the validity of the currently available mechanism of pathogenesis and suggested that the altered monoaminergic neurotransmitter system might not fully explain the molecular mechanism of GAD development, suggesting other pathophysiological factors might be involved in GAD. Recently, dysregulated immune systems have attracted great interest as an important pathophysiological factor for the development of GAD [4, 6–8]. Several clinical and preclinical studies suggest a link between the altered immune system and GAD pathology. Preclinical studies in mice also demonstrated that administration of pro-inflammatory cytokines (including IL-1β, TNF-α, and IL-6) in mice resulted in anxiety-like behaviors that were attenuated or normalized after injecting either anti-inflammatory cytokines or antagonists for the concerned cytokines [9–13]. A recent prospective cohort study conducted by Hou et al., (2019) demonstrated that administration of selective serotonin reuptake inhibitors (escitalopram or sertraline) resulted in a significant reduction in peripheral pro-inflammatory cytokines, and the authors suggested that the anxiolytic effects of these SSRIs might partly be based on their acute anti-inflammatory activities [14], implicating a significant association between dysregulated peripheral immune systems and GAD development. The development of anxiety-like symptoms in IL-4 gene knock-out mice, reduced levels of IL-4 in anxious mice, and the significant attenuation of anxiety-like behaviors following IL-4 injection demonstrated a positive association between anti-inflammatory cytokines, IL-4 levels, and anxiety pathology [15–18]. This immune hypothesis of GAD development is further potentiated by findings from several clinical studies that reported that GAD patients showed significantly higher levels of pro-inflammatory cytokines ( IL-1Ra, IL-1, IL-6, TNF-α, etc.) compared to healthy controls (HCs) [19–28] along with decreased levels of anti-inflammatory cytokines, including IL-4 and IL-10 [25]. Besides, pro-inflammatory cytokines such as TNF-α, and IL-6 were significantly associated with anxiety scores [29]. Consistent with this, a randomized clinical trial in humans demonstrated that LPS administration resulted in enhanced anxiety scores, and the authors suggested a significant correlation between pro-inflammatory cytokine levels and anxiety severity [30]. LPS-mediated microglia activation causes enhanced release of excessive pro-inflammatory cytokines in the basolateral amygdala, which ultimately leads to neuroinflammation in mice, resulting in the development of anxiety and depression-like behaviors by modulating neuronal plasticity. The authors found that anxiety pathogenesis was due to the excessive release of excitatory neurotransmitter glutamate from presynaptic axonal terminals of the prefrontal cortex, leading to neuroplasticity [31]. However, some studies reported either no significant variation in pro-inflammatory or anti-inflammatory cytokine serum levels between GAD patients and HCs [32] or that pro-inflammatory cytokines including IL-1, IL-2, and IL-6 were significantly reduced in GAD patients than HCs [33, 34]. This discrepancy in altered levels of inflammatory cytokines across clinical studies necessitates a further examination of the role of these cytokines in GAD pathophysiology.

Interleukin-2 (IL-2) is one of the major pro-inflammatory cytokines implicated in T cell activation, proliferation, and differentiation and is thus linked to excessive neuro-inflammatory processes [35]. IL-2 has been shown to impair synaptic plasticity and cause neuroinflammation, which ultimately leads to neuronal damage in neurocircuits associated with fear and anxiety signal transduction. IL-2 was also reported to act as a potent modulator of NMDA and kainite-mediated excitability in mesolimbic or mesostriatal systems [36–38] and thus affect neuroplasticity. As IL-2 was found to be positively associated with major depressive disorder [38, 39], probably, IL-2 might also be correlated with anxiety disorders like GAD, as MDD and GAD are highly co-morbid themselves and thus might share common pathophysiological factors. Recently, a preclinical study conducted by Gilio et al., (2022) observed that IL-2 administration in experimentally healthy mice triggered marked anxiety and depression-like behaviors, and the authors suggested that inhibition of GABA-mediated synaptic inhibitory neurotransmission was involved in the pathology of anxiety [40].

Interleukin-10 (IL-10) is one of the major anti-inflammatory cytokines that is secreted from Treg cells, Th2 cells, CD4 + T cells, CD8 + T cells, monocytes, macrophages, dendritic cells, B cells, neutrophils in the peripheral nervous system, and from microglia, astrocytes in the central nervous system (CNS) [41]. IL-10 signaling triggers anti-inflammatory, immunosuppressive, and immunoregulatory activities, including downregulating the production and secretion of pro-inflammatory cytokines and chemokines from activated macrophages, neutrophils, mast cells, Th1 cells, and DCS, decreasing the expression of MHC class II and co-stimulatory molecules on macrophages, and thereby suppressing the antigen presentation capacity of APCS [42–46]. In the CNS, it inhibits the production of such cytokines and chemokines by activated microglia and thereby counteracts cellular and tissue damage in response to excessive neuroinflammation [47, 48]. IL-10 has also been shown to stimulate axonal regeneration and activate wound healing through tissue repair [48]. Research also indicates its role as an inhibitor for microglial hyperactivation in response to LPS-induced inflammatory stimulus [49]. Based on its anti-inflammatory and immunoregulatory functions, researchers suggested an intricate role for IL-10 in several auto-immune and neuropsychiatric disorders. For example, Mesquita et al., (2008) observed that IL-10 KO mice developed markedly enhanced depressive-like behavior, which was attenuated after IL-10 administration, and that overexpression of IL-10 resulted in reduced depressive behaviors in mice [50]. Moreover, administration of IL-10 into rats attenuated the pro-inflammatory cytokine IL-1β-induced anxiety-like symptoms in male rats [10], demonstrating that IL-10 possesses anxiolytic activities. Preclinical research using an experimental animal model also suggests that the observed anxiolytic effect of several anti-anxiety drugs, including 3’-deoxyadenosine (3’-dA), imipramine, fluoxetine, and chlordiazepoxide, stems from their ability to upregulate anti-inflammatory cytokine (IL-4, IL-10) expression in the prefrontal cortex and locus coeruleus and simultaneous down-regulation of proinflammatory cytokine gene expression, leading to a correction of the imbalance between proinflammatory and anti-inflammatory states [51, 52]. Though several preclinical studies suggest a potential link between IL-10 levels and anxiety disorder, there is a scarcity of clinical studies aimed at evaluating such an association between IL-10 and GAD development [10].

Currently, there is no objective and cost-effective diagnostic or prognostic biomarker for GAD, which poses challenges in early diagnosis or risk prediction and leads to misdiagnosis or underdiagnosis, hampering the proper management of the disease. Currently available diagnostic tools, including self-reported symptoms and scoring severity based on the patient’s response to the 7-item questionnaire (GAD-7 scores), are subjective. Though neuroimaging techniques such as positron emission tomography (PET) and functional MRI can be used for the proper and objective diagnosis of GAD, due to their high cost and sophistication or complexities, these diagnostic tools are not suitable for either mass-level screening or are not easy to conduct multiple times to monitor the disease progression or therapeutic drug response. As such, the investigation of cost-effective objective biomarkers for GAD is one of the major focuses of current research on GAD. Finding a suitable biomarker is essential for early diagnosis and initiating psychotherapy and pharmacotherapy as early as possible [3]. Several studies were performed investigating the potential association between altered pro-inflammatory cytokines or anti-inflammatory cytokines and the pathogenesis of GAD. However, the actual role of inflammatory cytokines in GAD patients is not well explained. Therefore, the present study aims to explore the role of pro-inflammatory cytokines (IL-2) and anti-inflammatory cytokines (IL-10) in the pathophysiology and development of GAD. Also, we aim to find the potential associations of IL-2 and IL-10 with the severity of GAD patients. We believe the present study results would help to understand the pathophysiology and development of GAD.

## Methods

### Study population

We recruited 88 participants for this case-control study (50 GAD patients and 38 HCs matched by age and sex). Patients were collected from the Department of Psychiatry, Bangabandhu Sheikh Mujib Medical University Hospital, Dhaka, Bangladesh, and HCs from nearby areas of Dhaka city. A professional psychiatrist diagnosed patients and evaluated HCs based on DSM-5 criteria. We applied a 7-item GAD scale to assess the severity of anxiety symptoms [53]. The total scores range from 0 to 21, and it classifies the anxiety severity into four categories: minimal anxiety (0–4 scores), mild anxiety (5–9 scores), moderate anxiety (10–14 scores), and severe anxiety (15–21 scores). We excluded subjects with a co-morbidity of other psychiatric disorders, such as MDD, panic disorder, post-traumatic stress disorder, and social phobia, from the study. Additional exclusion criteria for participants were chronic liver and kidney diseases, infectious diseases, and alcohol or substance abuse. We also excluded patients who were exposed to anxiolytics or antidepressant medications within at least two weeks prior to the study that might have an impact on cytokine levels. We recorded the sociodemographic profile of the study population using a pre-designed questionnaire. The objectives of the study were explained to each participant, and informed written consent was obtained from them before their participation in this study. The study was conducted in accordance with the Declaration of Helsinki.

### Blood sample collection and serum isolation

A 5 ml blood sample was collected from the cephalic vein of each participant. The blood samples were kept at room temperature for 1 hour to ensure coagulation and were then subjected to centrifugation at 3000 rpm for 15 minutes at room temperature to collect serum samples. The collected serum was then placed in the Eppendorf tube and stored at -80 °C until further analysis.

### Estimation of serum cytokine levels

We estimated the serum levels of IL-2 and IL-10 by ELISA methods (Boster Bio, USA). We followed the manufacturer’s protocol for the ELISA assays. At first, we added 100 µl of standard cytokine solution, samples, and controls to each well of a pre-coated 96-well microplate. The microplates were covered with a plate sealer and incubated for 90 min at 37⁰C. After that, the cover was removed, and the liquid in each well was discarded. Subsequently, 100 µl of biotinylated anti-IL-2 antibody or anti-IL-10 antibody was incorporated into each well and incubated for 60 min at 37⁰C. After discarding the liquid from each well and washing it three times with 300 µl of wash buffer, 100 µl of avidin-biotin-peroxidase complex was added to each well, and the microplate was then again incubated for 30 min at 37⁰C. After the incubation period, the liquid was again discarded, and the plate was washed again with 300 µl of wash buffer five times. Following the addition of 90 µl color-developing reagent (TMB) into each well, the plate was incubated in a dark place for 30 min at RT, followed by the addition of 90 µl of stop solution to each well to stop the reaction process. We measured the absorbance with a microplate reader at 450 nm. We calculated the cytokine levels using standard curves and expressed them as pg/ml.

### Data presentation and statistical analysis

GraphPad Prism (version 8.0.1) and Statistical Package for the Social Sciences (version 24.0) were used for data analysis. We used descriptive statistics to find the variations in sociodemographic profiles and clinical characteristics between the groups. A T-test and a Chi-square test were employed to determine the statistical level of significance between the mean differences for variables across patients versus HC groups in the case of continuous variables and categorical variables, respectively. We used boxplot graphs for comparisons of analyzed cytokines between patients and HCs. We also generated scatter plot graphs for several clinical variables in GAD patients to show the correlations among the clinical parameters. A correlation analysis was performed to assess the potential association between several demographic and clinical variables in GAD patients. Receiver operating characteristics (ROC) analysis was conducted to determine the diagnostic efficacy of serum IL-2 or IL-10 levels in discriminating GAD patients from HCs. In all cases, statistical significance was considered at *p* < 0.05.

## Results

### Sociodemographic characteristics of the study population

The sociodemographic characteristics of the study population are presented in Table 1. The GAD patients and HCs were similar in terms of their age, sex, and BMI. Among the participants, about 60% were male and from urban areas. The majority of patients (60.00%) and HCs (68.42%) were unmarried. There was no significant variation between patients and HCs for their education level, occupation, economic status, or smoking status. In contrast, there was a difference between patients and HCs for their family history and previous history of the disease. In GAD patients, 20.00% had a family history, and 40.00% had a previous history of the disease.

**Table 1:** Socio-demographic characteristics of the study population

Parameters	GAD patients (*n* = 50) Mean ± SEM	Healthy controls (*n* = 38) Mean ± SEM	*p* value
Age in years	31.04 ± 1.52	30.66 ± 2.04	0.878
18–25	20 (40.00%)	14 (36.84)	
26–35	19 (38.00%)	16 (42.11)	
36–45	3 (6.00%)	2 (5.26)	
46–60	8 (16.00%)	6 (15.79)	
Sex			0.974
Male	30 (60.00%)	23 (60.50%)	
Female	20 (40.00%)	15 (39.50%)	
Marital status			0.504
Married	20 (40.00%)	12 (31.58%)	
Unmarried	30 (60.00%)	26 (68.42%)	
BMI (kg/m^2^)	23.49 ± 0.57	24.70 ± 0.71	0.179
Below 18.5 (CED)	3 (6.00%)	4 (10.53%)	
18.5–25 (normal)	31 (62.00%)	15 (39.47%)	
Above 25 (obese)	16 (32.00%)	19 (50.00%)	
Education level			0.244
Illiterate	4 (8.00%)	0 (0.00%)	
Primary level	7 (14.00%)	6 (15.79%)	
Secondary level	5 (10.00%)	1 (2.63%)	
Higher Secondary level	14 (28.00%)	14 (36.84%)	
Graduate and above	20 (40.00%)	17 (44.74%)	
Occupation			0.861
Housewife	12 (24.00%)	6 (15.79%)	
Business	5 (10.00%)	3 (7.89%)	
Unemployed/pensioner	14 (28.00%)	13 (34.21%)	
Student	12 (24.00%)	11 (28.95%)	
Others	7 (14.00%)	5 (13.16%)	
Economic status			0.477
High	2 (4.00%)	2 (5.26%)	
Medium	38 (76.00%)	32 (84.21%)	
Low	10 (20.00%)	4 (10.53%)	
Smoking history			0.131
Nonsmoker	43 (86.00%)	37 (97.37%)	
Smoker	7 (14.00%)	1 (2.63%)	
Residence area			0.830
Rural	21 (42.00%)	15 (39.47%)	
Urban	29 (58.00%)	23 (60.53%)	
Family history of GAD			**0.004**
Yes	10 (20.00%)	0 (0.00%)	
No	40 (80.00%)	38 (100.00%)	
Previous history of GAD			**< 0.001**
Yes	20 (40.00%)	0 (0.00%)	
No	30 (60.00%)	38 (100.00%)	

Abbreviations: BMI: body mass index; CED: chronic energy deficiency; GAD: generalized anxiety disorder; SEM: standard error mean; Data is considered to be statistically significant *p* < 0.05

### Clinical characteristics and laboratory findings

**Fig. 1:**
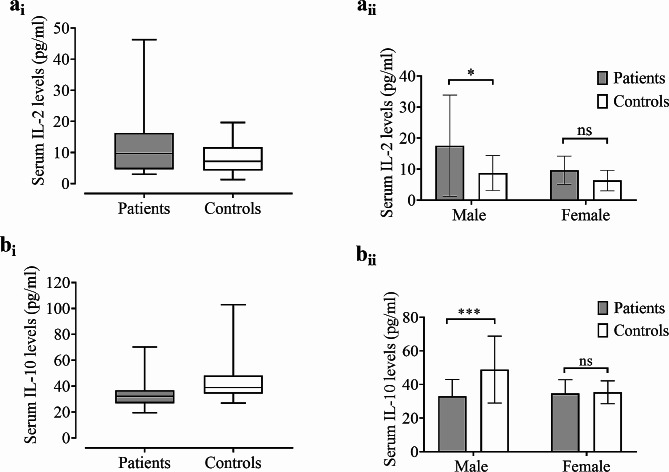
Distribution of serum IL-2 (**a**_**i**_) and IL-10 (**b**_**i**_) levels in GAD patients and healthy controls. Comparison of IL-2 and IL-10 levels between GAD patients and their counterparts in control subjects are showed in **a**_**i**_ and **b**_**i**_. Comparison of IL-2 and IL-10 levels between male or female GAD patients and their counterparts in control subjects are presented in **a**_**ii**_ and **b**_**ii**_

Clinical characteristics and laboratory analysis results are presented in Table 2. GAD patients displayed markedly higher serum levels of IL-2 (14.81 ± 2.88 pg/ml) compared to HCs (8.08 ± 1.10 pg/ml), and the difference reached the statistically significant level (*p* = 0.037, two-tailed unpaired t-test) (Table 2; Fig. 1). Though male GAD patients exhibited markedly higher levels of IL-2 compared to male HCs (*p* = 0.048), there was no significant variation in IL-2 levels between female patients and female HCs (*p* > 0.05) (Fig. 1). Though some 1.8-fold higher IL-2 serum levels were observed in male GAD patients compared to female GAD patients, the difference did not reach the statistical significance level (*p* = 0.198, two-tailed unpaired t-test). In contrast to the results obtained for IL-2, IL-10 showed a statistically significant (*p* < 0.001) reduction in GAD patients (33.69 ± 1.37 pg/ml) compared to HCs (44.12 ± 3.16 pg/ml) (Fig. 1). Similar to the results obtained for IL-2, IL-10 levels showed a statistically significant difference between patients versus HCs when male people were considered (Fig. 1). In contrast, there was no significant variation in IL-10 levels between female GAD patients and female HCs (*p* > 0.05).

**Table 2:** Clinical characteristics and laboratory findings of the study population

Parameters	GAD patients (*n* = 50) Mean ± SEM	Healthy controls (*n* = 38) Mean ± SEM	*p* value
DSM-5 scores	9.62 ± 0.17	-	-
GAD-7 scores	13.08 ± 0.57	-	-
IL-2 (pg/ml)	14.81 ± 2.88	8.08 ± 1.10	**0.037**
Male	17.56 ± 4.21	8.73 ± 1.40	**0.048**
Female	9.67 ± 1.61	6.33 ± 1.36	0.156
IL-10 (pg/ml)	33.69 ± 1.37	44.12 ± 3.16	**0.001**
Male	33.08 ± 1.88	48.90 ± 4.45	**< 0.001**
Female	34.70 ± 1.98	35.42 ± 2.06	0.810

Abbreviation: DSM-5: diagnostic and statistical manual for mental disorders; 5th edition: GAD: generalized anxiety disorder; GAD-7: generalized anxiety disorder 7-item scores; SEM: standard error mean; IL-2: interleukin-2; IL-10: interleukin-10. Two-tailed unpaired student’s *t* test was applied for evaluating statistical significance level between mean difference between cases and controls and *p* < 0.05 was statistically significant

### Correlation analysis among different study parameters

**Fig. 2:**
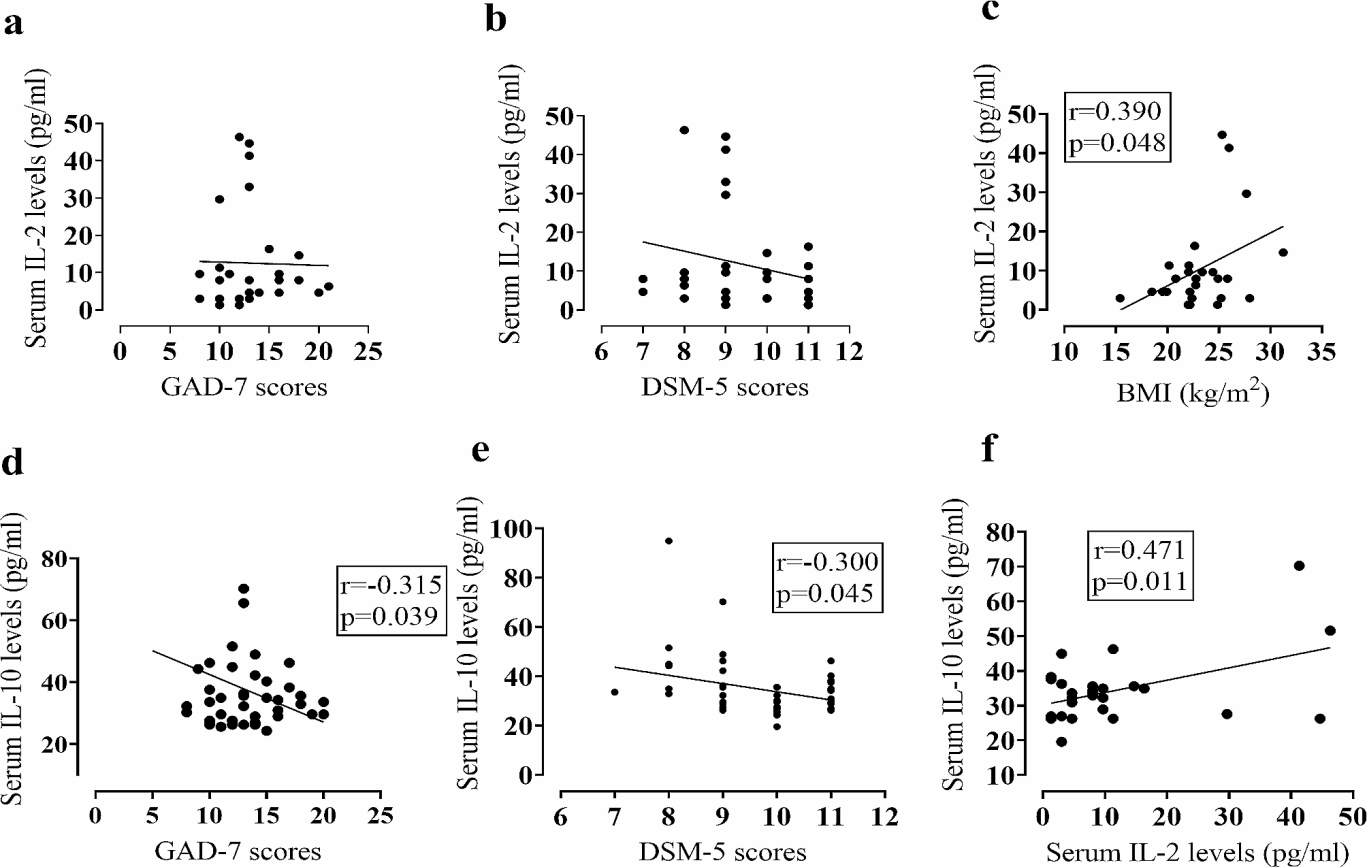
Scatter plot graphs for several clinical variables of GAD patients showing existence or absence of correlation between the clinical parameters. Scatter plot for serum IL-2 levels versus GAD-7 scores (**a**) or DSM-5 scores (**b**) expressing no significant association between IL-2 and both clinical parameters. Scatter plot graphs showing significant association between IL-2 levels and BMI (**c**), IL-10 levels and GAD-7 scores (**d**), IL-10 levels and DSM-5 scores and IL-10 and IL-2 levels (**f**)

We then performed a series of correlation analyses to investigate the association of altered cytokine serum levels with several demographic and clinical variables, such as age, BMI, DSM-5, and GAD-7 scores (Table 3). Serum IL-2 levels did not show any positive or negative association with either DSM-5 or GAD-7 scores (*p* > 0.05), suggesting that despite its significant enhancement in GAD patients compared to HCs, IL-2 may not associate with GAD pathophysiology. We also observed no significant association between the ages of the patients and IL-2 serum levels. In contrast, the IL-2 levels of GAD patients maintained a significant and positive correlation with BMI levels of patients (*r* = 0.390, *p* < 0.05) which is consistent with the intricate relationship between body mass and enhanced pro-inflammatory responses. Contrary to the results obtained for IL-2, reduced serum IL-10 levels maintained a significant but negative association with both DSM-5 scores (*r*=-0.300, *p* = 0.045) and GAD-7 scores (*r*=-0.315, *p* = 0.039), implicating that altered IL-10 levels are linked to GAD development or pathogenesis. However, the age and BMI levels of GAD patients failed to show any positive or negative association with IL-10 serum levels. Analysis also showed a significant and strong positive association between IL-2 and IL-10 serum levels (*r* = 0.471, *p* = 0.011) in GAD patients, which might be due to the compensatory enhancement of anti-inflammatory cytokine, IL-10 in response to elevated pro-inflammatory cytokine, IL-2 levels. Also, we displayed these correlations among several clinical variables of GAD patients by scatter plot graphs (Fig. 2).

**Table 3:** Correlation analysis among different analyzed parameters

Parameters	Correlations	
	*r*	*p*
Age and IL-2	0.142	0.454
Age and GAD-7 scores	0.043	0.763
Age and IL-10	-0.116	0.445
BMI and GAD-7 scores	0.061	0.675
BMI and IL-2	0.390	**0.048**
BMI and IL-10	0.092	0.540
IL-2 and GAD-7 scores	-0.022	0.908
IL-10 and GAD-7 scores	-0.315	**0.039**
IL-2 and DSM-5 scores	-0.225	0.230
IL-10 and DSM-5 scores	-0.300	**0.045**
IL-2 and IL-10	0.471	**0.011**

Abbreviation: BMI: body mass index; GAD-7: generalized anxiety disorder 7-item scores

### Receiver operating characteristic curve analysis

**Fig. 3:**
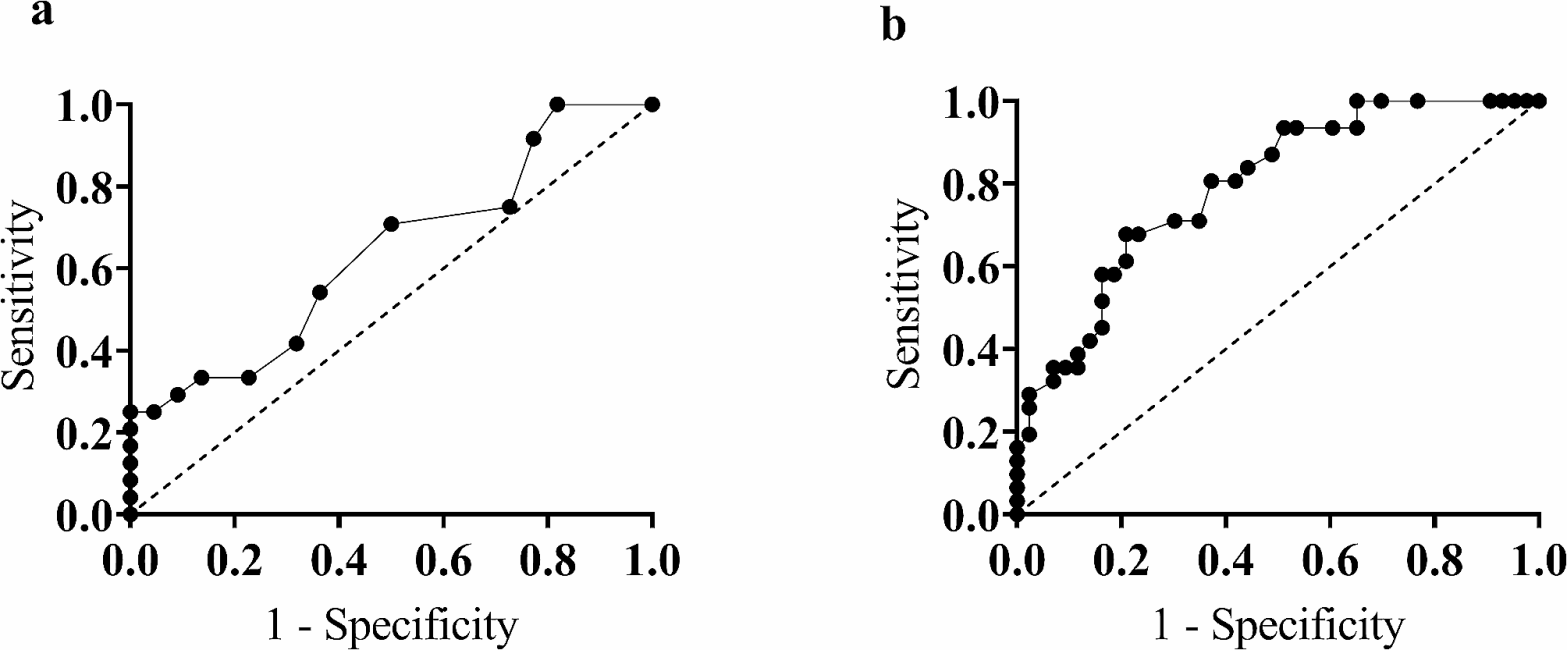
Receiver operating characteristic curve (ROC) for serum IL-2 (**a**) and IL-10 levels (**b**)

Serum IL-10 measurement showed a good performance in differentiating GAD patients from HCs, which was evidenced by its significantly higher area under the curve (AUC) value of 0.793 (*p* < 0.001) with 80.65% sensitivity and 62.79% specificity at a cut-off value of 33.93 pg/ml, in which the cytokine levels below this point indicate disease states (Table 4; Fig. 3). ROC analysis of serum IL-2 levels failed to discriminate GAD patients from HCs as the AUC value was below the acceptable range (AUC: 0.640; *p* = 0.108) with 54.17% sensitivity and 68.18% specificity at a cut-off value of 8.83 pg/ml) (Fig. 3; Table 4).

**Table 4:** Receiver operating characteristic curve analysis of serum IL-2 and IL-10 levels as discriminators between GAD patients and healthy controls

Cytokine	Cut-off value (pg/ml)	AUC	95% CI		Sensitivity (%)	Specificity (%)	*p* value
			Lower Limit	Upper Limit			
IL-2	8.83	0.640	0.478	0.798	54.17	68.18	0.108
IL-10	33.93	0.793	0.693	0.893	80.65	62.79	**< 0.001**

Abbreviation: AUC: area under the curve; CI: confidence interval; GAD: generalized anxiety disorder; IL-2: interleukin-2; IL-10: interleukin-10

## Discussion

To the best of our knowledge, this is the first case-control study to investigate the potential association between the pathophysiology of GAD and the pro-inflammatory cytokine, IL-2, and the anti-inflammatory cytokine, IL-10, among the Bangladeshi population. We observed that IL-10 serum levels were significantly lower in GAD patients than in HCs, and this reduction was found to be significantly but negatively associated with both DSM-5 scores and GAD-7 scores, demonstrating potential involvement of this anti-inflammatory cytokine in disease severity and symptoms. Our results of a significant reduction in IL-10 levels in GAD patients are in good agreement with those observed in other studies [23, 25]. In contrast, our results diverge from those reported by others [33, 54] who either reported no significant variation in IL-10 levels between GAD patients and HCs or that IL-10 levels were enhanced in GAD patients compared to HCs. ROC analysis also demonstrated the good predictive value of IL-10 serum measurement in discriminating diseased patients from HCs, suggesting that IL-10 serum level might be a potential biomarker for diagnosis, anti-anxiety drug response monitoring, or disease progression monitoring. Recently, Hou et al. (2019) demonstrated that peripheral serum levels of the pro-inflammatory cytokine IL-6 could be used to monitor the treatment response of SSRIs in GAD [14]. Similarly, IL-10 might be used as a marker for therapeutic drug monitoring in GAD. However, further longitudinal studies are required to find any causal relationship between IL-10 and disease severity or pathogenesis. On the other hand, serum IL-2 levels were significantly elevated in GAD patients compared to HCs, but they failed to demonstrate any significant association with either DSM-5 scores or GAD-7 scores in Pearson correlation analysis, implying that IL-2 levels might not be associated with the pathophysiology and development of GAD. Consistent with this, ROC analysis showed that IL-2 levels have no significant diagnostic efficacy in differentiating GAD patients from HCs. Further analysis with a larger population size is required to explore the role of IL-2 in the context of GAD severity. Our results are consistent with those reported by Tang et al. (2018), who also observed that GAD patients exhibited significantly higher serum levels of IL-2 compared to HCs [19]. However, our results are not in agreement with those reported by others who observed either no significant variation in IL-2 levels [54] or a significant reduction in GAD patients compared to HCs [25, 33, 34, 55]. We also observed a significant positive correlation between IL-2 and IL-10 levels in GAD patients, which indicates a compensatory mechanism [56].

Our study provides some valuable insights into the complex and intricate relationship between the dysregulated immune system and GAD. The observed reduction in IL-10 levels in GAD patients in our study suggests a potential immunoregulatory imbalance in GAD, with IL-10 playing a role in modulating anxiety severity. The lack of a significant association between IL-2 serum levels and anxiety severity highlights the nuanced nature of immune dysregulation in GAD, warranting further exploration into the specific mechanisms involved. Elevated levels of pro-inflammatory cytokine, IL-2, and decreased levels of anti-inflammatory cytokine, IL-10, in GAD patients compared to HCs indicate that GAD individuals of the Bangladeshi cohort are characterized by heightened inflammatory responses derived from the imbalance between pro-inflammatory and anti-inflammatory states. Our study finding provides further support for the cytokine hypothesis of anxiety disorder, which proposes that pro-inflammatory cytokine-mediated neuroinflammatory processes can lead to anxiety symptoms or behaviors by downregulating serotonin biosynthesis or enhancing the reuptake of serotonin, resulting in an altered serotonergic neurotransmitter system in the CNS [15]. The observed significant negative correlation between IL-10 and DSM-5 scores or GAD-7 scores suggests that lowering IL-10 levels might be involved in the pathogenesis of GAD. One of the major implications of our study findings is that IL-10 signaling might be targeted to explore potential novel immunological/immunomodulatory therapies against GAD. The diminished IL-10 levels and their negative correlation with GAD severity suggest a potential avenue for therapeutic intervention. IL-10 might also be used as an anti-inflammatory adjunctive therapy with other pharmacotherapies including SSRIs/SNRIs. However, at this moment, we don’t know the exact mechanism by which lowered levels of IL-10 are linked to higher anxiety severity in GAD patients.

As IL-10 has anti-inflammatory and immunoregulatory activities such as suppression of production of pro-inflammatory cytokines (IL-1β, IL-6, and TNF-α) from microglia and astrocytes, reduction in IL-10 levels in GAD patients in our study led to an imbalance between pro-inflammatory and anti-inflammatory states and resulted in enhanced pro-inflammatory responses, which might be the cause of enhanced anxiety symptoms as inflammatory cytokine-mediated neuroinflammation was reported to be linked with disrupted monoaminergic neurotransmission in the brain. Besides, elevated levels of IL-10 were shown to attenuate anxiety-like behaviors by modulating GABAergic neurotransmission in the amygdala (Patel et al., 2021). IL-10 was also reported to display some neuroprotective activities and has been shown to inhibit neuronal apoptosis and promote neurite outgrowth, axonal outgrowth, and synapse formation in the brain by the JAK1-STAT3 signaling pathway [57]. In a preclinical study, IL-4 has been shown to cause the shifting of microglia and macrophages from pro-inflammatory to anti-inflammatory neuroprotective phenotypes characterized by excessive production of arginase-1 and PPARγ receptor expression in microglia and macrophage and thereby attenuating brain-injury-mediated anxiety by inhibiting neuronal loss and nerve tracts in the limbic system [58]. A similar mechanism might be involved in IL-10-mediated anxiety symptom improvement in GAD patients. Further research is required to unravel the exact mechanisms of IL-10-mediated anxiety symptom attenuation in GAD patients.

In terms of diagnostic marker development, as IL-10 serum level measurement demonstrated good performance in discriminating GAD patients from HCs and as IL-10 levels maintained a significant and negative correlation with disease severity, IL-10 serum level raised the possibility of being an objective biomarker for GAD. However, the diagnostic efficacy of this cytokine should be investigated thoroughly using a range of longitudinal studies. Despite this, at this time we can conclude that IL-10 might be used as a risk indicator for assessment of susceptibility to anxiety disorder, resulting in early detection of the disease and prompting the initiation of intervention strategies. This early detection will reduce treatment costs and decrease the prevalence and morbidity associated with this chronic disorder.

The strength of our study is that we designed a set of inclusion and exclusion criteria for the recruitment of participants and followed those criteria in such a way that homogenous population data could be obtained. The strict study design helped us enormously to minimize the potential impact of several confounding variables, including age, sex, BMI, co-morbid diseases, and immunomodulatory drugs, on cytokine levels. However, our study also has some limitations that should be acknowledged. The major limitation of this study is the smaller sample size. We recruited 50 patients and 38 HCs, which does not represent the whole Bangladeshi demographic. It would be better if we could enroll an equal number of cases and controls. For example, we observed that cytokine levels maintained a statistically significant difference between male GAD patients and male HCs. In contrast, no significant variation in cytokine levels was observed when female data were considered. As we have included more male participants (60%) than female participants (40%), the lower sample size of female participants might generate a higher background noise, resulting in lower statistical power, warranting further studies recruiting a larger population size to investigate sex-specific differences in cytokine levels in GAD patients. Our case-control study design is inherently correlational and thus not able to evaluate the causal relationship between altered cytokine levels and GAD. So, at this moment, we cannot conclude whether the altered levels of serum cytokines are the causes of anxiety development or just the outcome of pathophysiological changes.

Longitudinal studies are required to investigate whether altered cytokine levels precede GAD or if it’s just a mere reflection of GAD pathology. Though we have restricted the impacts of several co-variates, other confounding variables, including genetic polymorphism in cytokine genes, the effect of lifestyle or xenobiotics, and dietary habits, were not considered, which might have modulatory effects on serum cytokine levels.

## Conclusion

The study provides valuable insights for understanding the pathogenesis of GAD. Despite having elevated IL-2 levels in GAD patients compared to HCs, it failed to demonstrate a significant association with anxiety severity as assessed by GAD-7 scores. In contrast, serum IL-10 levels were significantly reduced in GAD patients compared to HCs and showed a significant negative correlation with anxiety severity, implicating a potential link with the GAD pathophysiology. Our results support the immune hypothesis of GAD development. Our study findings also suggest that IL-10 serum level measurement might offer an objective blood-based biomarker or risk assessment indicator for GAD. We recommend further research employing a larger population size and homogenous data from different areas of Bangladesh to confirm our study findings.

## Data Availability

All the relevant data and information will be available from the corresponding author upon reasonable request.

## Abbreviations

AUC: The curve
BMI: Body mass index
CED: Chronic energy deficiency
CI: Confidence interval
CNS: Central nervous system
DSM-5: Diagnostic and statistical manual for mental disorders, 5th edition
ELISA: Enzyme-linked immunosorbent assay
GAD: Generalized anxiety disorder
GAD-7: Generalized anxiety disorder 7-item scores
HC: Healthy control
IL-2: Interleukin-2
IL-10: Interleukin-10
ROC: Receiver operating characteristic
SEM: Standard error mean
SPSS: Statistical package for social science

## References

[1] Fagan HA, Baldwin DS (2023). Pharmacological treatment of generalised anxiety disorder: current practice and future directions. Expert Rev Neurother.

[2] Strawn JR, Geracioti L, Rajdev N, Clemenza K, Levine A (2018). Pharmacotherapy for generalized anxiety disorder in adult and pediatric patients: an evidence-based treatment review. Expert Opin Pharmacother.

[3] Maron E, Nutt D (2017). Biological markers of generalized anxiety disorder. Dialogues Clin Neurosci.

[4] Costello H, Gould RL, Abrol E, Howard R (2019). Systematic review and meta-analysis of the association between peripheral inflammatory cytokines and generalised anxiety disorder. BMJ Open.

[5] Ansara ED (2020). Management of treatment-resistant generalized anxiety disorder. Ment Health Clin.

[6] Michopoulos V, Powers A, Gillespie CF, Ressler KJ, Jovanovic T (2017). Inflammation in fear- and anxiety-based disorders: PTSD, GAD, and beyond. Neuropsychopharmacology.

[7] Renna ME, O’Toole MS, Spaeth PE, Lekander M, Mennin DS. The association between anxiety,traumatic stress, and obsessive-compulsive disorders and chronic inflammation: A systematic review and meta-analysis. Depress Anxiety. 2018;;35(11):1081–1094. doi: 10.1002/da.22790. 10.1002/da.22790 30199144

[8] Hou R, Baldwin DS (2012). A neuroimmunological perspective on anxiety disorders. Hum Psychopharmacol.

[9] Zhu CB, Lindler KM, Owens AW, Daws LC, Blakely RD, Hewlett WA (2010). Interleukin-1 receptor activation by systemic lipopolysaccharide induces behavioral despair linked to MAPK regulation of CNS serotonin transporters. Neuropsychopharmacology.

[10] Munshi S, Parrilli V, Rosenkranz JA (2019). Peripheral anti-inflammatory cytokine Interleukin-10 treatment mitigates interleukin-1β - induced anxiety and sickness behaviors in adult male rats. Behav Brain Res.

[11] Bercik P, Verdu EF, Foster JA, Macri J, Potter M, Huang X (2010). Chronic gastrointestinal inflammation induces anxiety-like behavior and alters central nervous system biochemistry in mice. Gastroenterology.

[12] Gentile A, Fresegna D, Musella A, Sepman H, Bullitta S, De Vito F et al. Interaction between interleukin-1β and type-1 cannabinoid receptor is involved in anxiety-like behavior in experimental autoimmune encephalomyelitis. J Neuroinflammation. 2016;13(1):231. Published 2016 Sep 2. 10.1186/s12974-016-0682-8. 10.1186/s12974-016-0682-8 PMC5009553 27589957

[13] Haji N, Mandolesi G, Gentile A, Sacchetti L, Fresegna D, Rossi S (2012). TNF-α-mediated anxiety in a mouse model of multiple sclerosis. Exp Neurol.

[14] Hou R, Ye G, Liu Y, Chen X, Pan M, Zhu F (2019). Effects of SSRIs on peripheral inflammatory cytokines in patients with generalized anxiety disorder. Brain Behav Immun.

[15] Quagliato LA, Nardi AE (2022). Cytokine profile in drug-naïve panic disorder patients. Transl Psychiatry.

[16] Lee HJ, Park HJ, Starkweather A, An K, Shim I (2016). Decreased Interleukin-4 release from the neurons of the Locus Coeruleus in response to immobilization stress. Mediators Inflamm.

[17] Gao T, Li B, Hou Y, Luo S, Feng L, Nie J (2019). Interleukin-4 signalling pathway underlies the anxiolytic effect induced by 3-deoxyadenosine. Psychopharmacology.

[18] Moon ML, Joesting JJ, Blevins NA, Lawson MA, Gainey SJ, Towers AE (2015). IL-4 knock out mice display anxiety-like Behavior. Behav Genet.

[19] Tang Z, Ye G, Chen X, Pan M, Fu J, Fu T (2018). Peripheral proinflammatory cytokines in Chinese patients with generalised anxiety disorder. J Affect Disord.

[20] Yang CJ, Liu D, Xu ZS, Shi SX, Du YJ (2017). The pro-inflammatory cytokines, salivary cortisol and alpha-amylase are associated with generalized anxiety disorder (GAD) in patients with asthma. Neurosci Lett.

[21] Vogelzangs N, Beekman AT, de Jonge P, Penninx BW (2013). Anxiety disorders and inflammation in a large adult cohort. Transl Psychiatry.

[22] Vieira MM, Ferreira TB, Pacheco PA, Barros PO, Almeida CR, Araújo-Lima CF (2010). Enhanced Th17 phenotype in individuals with generalized anxiety disorder. J Neuroimmunol.

[23] Hou R, Garner M, Holmes C, Osmond C, Teeling J, Lau L (2017). Peripheral inflammatory cytokines and immune balance in generalised anxiety disorder: case-controlled study. Brain Behav Immun.

[24] Copeland WE, Shanahan L, Worthman C, Angold A, Costello EJ (2012). Generalized anxiety and C-reactive protein levels: a prospective, longitudinal analysis. Psychol Med.

[25] Ferreira TB, Kasahara TM, Barros PO, Vieira MM, Bittencourt VC, Hygino J (2011). Dopamine up-regulates Th17 phenotype from individuals with generalized anxiety disorder. J Neuroimmunol.

[26] Bankier B, Barajas J, Martinez-Rumayor A, Januzzi JL (2008). Association between C-reactive protein and generalized anxiety disorder in stable coronary heart disease patients. Eur Heart J.

[27] Maes M, Song C, Lin A, De Jongh R, Van Gastel A, Kenis G (1998). The effects of psychological stress on humans: increased production of pro-inflammatory cytokines and a Th1-like response in stress-induced anxiety. Cytokine.

[28] Lu H, Yang Q, Zhang Y (2022). The relation of common inflammatory cytokines with anxiety and depression and their values in estimating cardiovascular outcomes in coronary heart disease patients. J Clin Lab Anal.

[29] Pitsavos C, Panagiotakos DB, Papageorgiou C, Tsetsekou E, Soldatos C, Stefanadis C (2006). Anxiety in relation to inflammation and coagulation markers, among healthy adults: the ATTICA study. Atherosclerosis.

[30] Lasselin J, Elsenbruch S, Lekander M, Axelsson J, Karshikoff B, Grigoleit JS (2016). Mood disturbance during experimental endotoxemia: predictors of state anxiety as a psychological component of sickness behavior. Brain Behav Immun.

[31] Zheng ZH, Tu JL, Li XH, Hua Q, Liu WZ, Liu Y (2021). Neuroinflammation induces anxiety- and depressive-like behavior by modulating neuronal plasticity in the basolateral amygdala. Brain Behav Immun.

[32] Mongan D, Raj SS, Föcking M, Byrne JF, Zammit S, Cannon M (2023). Associations between plasma inflammatory markers and psychotic disorder, depressive disorder and generalised anxiety disorder in early adulthood: a nested case-control study. Brain Behav Immun.

[33] Shen Z, Cui L, Mou S, Ren L, Yuan Y, Shen X (2022). Combining S100B and cytokines as neuro-inflammatory biomarkers for diagnosing generalized anxiety disorder: a proof-of-Concept Study based on machine learning. Front Psychiatry.

[34] Wagner EN, Strippoli MF, Ajdacic-Gross V, Gholam-Rezaee M, Glaus J, Vandeleur C (2020). Generalized anxiety disorder is prospectively Associated with decreased levels of Interleukin-6 and Adiponectin among individuals from the community. J Affect Disord.

[35] Ross SH, Cantrell DA (2018). Signaling and function of Interleukin-2 in T lymphocytes. Annu Rev Immunol.

[36] Ye JH, Tao L, Zalcman SS (2001). Interleukin-2 modulates N-methyl-D-aspartate receptors of native mesolimbic neurons. Brain Res.

[37] Ye JH, Zalcman SS, Tao L (2005). Kainate-activated currents in the ventral tegmental area of neonatal rats are modulated by interleukin-2. Brain Res.

[38] Suhee FI, Shahriar M, Islam SMA, Bhuiyan MA, Islam MR (2023). Elevated serum IL-2 levels are Associated with Major Depressive disorder: a case-control study. Clin Pathol.

[39] Köhler CA, Freitas TH, Maes M, de Andrade NQ, Liu CS, Fernandes BS (2017). Peripheral cytokine and chemokine alterations in depression: a meta-analysis of 82 studies. Acta Psychiatr Scand.

[40] Gilio L, Fresegna D, Gentile A, Guadalupi L, Sanna K, De Vito F (2022). Preventive exercise attenuates IL-2-driven mood disorders in multiple sclerosis. Neurobiol Dis.

[41] Carlini V, Noonan DM, Abdalalem E, Goletti D, Sansone C, Calabrone L (2023). The multifaceted nature of IL-10: regulation, role in immunological homeostasis and its relevance to cancer, COVID-19 and post-COVID conditions. Front Immunol.

[42] Fiorentino DF, Bond MW, Mosmann TR (1989). Two types of mouse T helper cell. IV. Th2 clones secrete a factor that inhibits cytokine production by Th1 clones. J Exp Med.

[43] Fiorentino DF, Zlotnik A, Mosmann TR, Howard M, O’Garra A (1991). IL-10 inhibits cytokine production by activated macrophages. J Immunol.

[44] Fiorentino DF, Zlotnik A, Vieira P, Mosmann TR, Howard M, Moore KW (1991). IL-10 acts on the antigen-presenting cell to inhibit cytokine production by Th1 cells. J Immunol.

[45] Bogdan C, Vodovotz Y, Nathan C (1991). Macrophage deactivation by interleukin 10. J Exp Med.

[46] Murray PJ (2005). The primary mechanism of the IL-10-regulated antiinflammatory response is to selectively inhibit transcription. Proc Natl Acad Sci U S A.

[47] Lobo-Silva D, Carriche GM, Castro AG, Roque S, Saraiva M (2016). Balancing the immune response in the brain: IL-10 and its regulation. J Neuroinflammation.

[48] Saraiva M, Vieira P, O’Garra A (2020). Biology and therapeutic potential of interleukin-10. J Exp Med.

[49] Shemer A, Scheyltjens I, Frumer GR, Kim JS, Grozovski J, Ayanaw S (2020). Interleukin-10 prevents pathological Microglia Hyperactivation following Peripheral Endotoxin Challenge. Immunity.

[50] Mesquita AR, Correia-Neves M, Roque S, Castro AG, Vieira P, Pedrosa J (2008). IL-10 modulates depressive-like behavior. J Psychiatr Res.

[51] Obuchowicz E, Bielecka AM, Paul-Samojedny M, Pudełko A, Kowalski J (2014). Imipramine and fluoxetine inhibit LPS-induced activation and affect morphology of microglial cells in the rat glial culture. Pharmacol Rep.

[52] Blatteau JE, de Maistre S, Lambrechts K, Abraini J, Risso JJ, Vallée N (2015). Fluoxetine stimulates anti-inflammatory IL-10 cytokine production and attenuates sensory deficits in a rat model of decompression sickness. J Appl Physiol (1985).

[53] Spitzer RL, Kroenke K, Williams JB, Löwe B (2006). A brief measure for assessing generalized anxiety disorder: the GAD-7. Arch Intern Med.

[54] Tofani T, Mannelli LD, Zanardelli M (2015). An immunologic profile study in drug-naive generalized anxiety non depressed patients: a pilot study. Eur Neuropsychopharmacol.

[55] Koh KB, Lee BK (1998). Reduced lymphocyte proliferation and interleukin-2 production in anxiety disorders. Psychosom Med.

[56] Inaba A, Tuong ZK, Zhao TX (2023). Low-dose IL-2 enhances the generation of IL-10-producing immunoregulatory B cells. Nat Commun.

[57] Chen H, Lin W, Zhang Y, Lin L, Chen J, Zeng Y (2016). IL-10 promotes neurite outgrowth and synapse formation in cultured cortical neurons after the oxygen-glucose deprivation via JAK1/STAT3 pathway. Sci Rep.

[58] Pu H, Wang Y, Yang T, Leak RK, Stetler RA, Yu F (2023). Interleukin-4 mitigates anxiety-like behavior and loss of neurons and fiber tracts in limbic structures in a microglial PPARγ-dependent manner after traumatic brain injury. Neurobiol Dis.

